# Experimental testing of a prototype cantilevered liquid-nitrogen-cooled silicon mirror

**DOI:** 10.1107/S1600577522010700

**Published:** 2023-01-01

**Authors:** G. Cutler, D. Cocco, B. Bentley, M. Cervantes, P. Chavez, J. Chrzan, S. DiMaggio, R. Hussey, J. Ilmberger, J. Lindsay, E. Lizotte, K. McCombs, S. Morton, G. Paulovits, K. Pearson, C. Redding, N. Smith, K. Tokunaga, D. Zehm, E. DiMasi, H. Padmore

**Affiliations:** a Lawrence Berkeley National Laboratory, Berkeley, California, USA; Uppsala University, Sweden

**Keywords:** cantilevered silicon mirrors, X-ray optics, mirror cooling, Advanced Light Source Upgrade

## Abstract

This report presents testing of a prototype cantilevered liquid-nitrogen-cooled silicon mirror. This mirror was designed to be the first mirror for the new soft X-ray beamlines to be built as part of the Advanced Light Source Upgrade. Test activities focused on fracture, heat transfer, modal response and distortion, and indicated that the mirror functions as intended.

## Abstract 2

Experimental testing of a liquid-nitrogen-cooled silicon mirror is reported. This testing covered fracture, heat transfer, modal response and distortion.

## 1. Introduction

**Figure 1:**
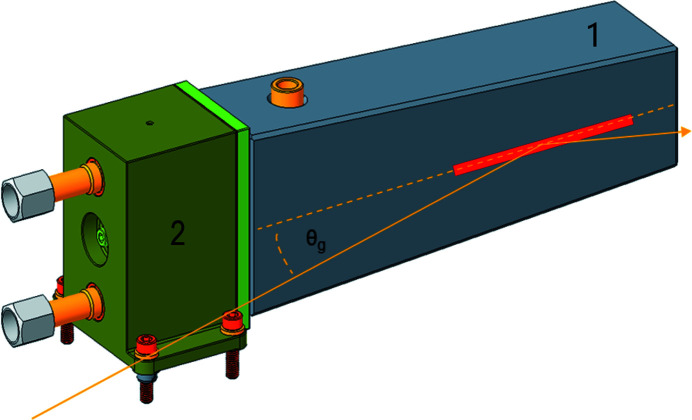
The silicon mirror substrate (1) is supported as a cantilever by the Invar coolant manifold (2). Liquid nitrogen flows through the manifold. The typical beam footprint is shown in red on the reflecting surface, along with the beam path with the grazing angle θ_g_ (in orange).

Here we describe the experience accumulated to date through testing the prototype mirror system we designed for the Advanced Light Source Upgrade (ALS-U) insertion device beamlines. This prototype, referred to internally at Lawrence Berkeley National Laboratory (LBNL) and in this report as the ‘cryocantilever,’ was designed for the high heat loads of the first beamline mirror (M1) and features a liquid-nitrogen-cooled silicon substrate clamped at one end to a manifold, which both cools and supports the substrate (Fig. 1 ▸). The cantilevered substrate differentiates this design from many existing synchrotron radiation beamline mirrors. The motivation for this cantilevered design was to minimize distortion of the optically significant region of the mirror substrate by isolating it from reaction forces applied to the substrate by the mounting system. By integrating the support and cooling the substrate on a single face of the manifold we also eliminated cooling ‘blocks’ (or additional parts in contact with the substrate) and the forces they would apply, and attenuate forces applied to the manifold by coolant lines. For a detailed description of the theory and calculations behind the design the reader is referred to the work by Cutler *et al.* (2020 ▸).

The testing described in this report was intended to address concerns related to the cantilevered design and uncover any problems that need to be addressed in the final ALS-U design. In this contribution, the first two sections summarize the design and fabrication of the prototype. Each of the remaining four sections are dedicated to a topic of particular concern for a cantilevered silicon mirror: fracture, heat transfer, modal response and distortion.

## 2. Fabrication and assembly of the prototype

The mirror substrate was fabricated and polished by InSync, Inc. of Albuquerque, New Mexico, USA. After machining, the substrate was etched to remove residual stresses and surface defects and then one side of the substrate was polished flat to a height error of 8 nm RMS and a roughness of 0.8 Å (J. Metz, personal communication).

**Figure 2:**
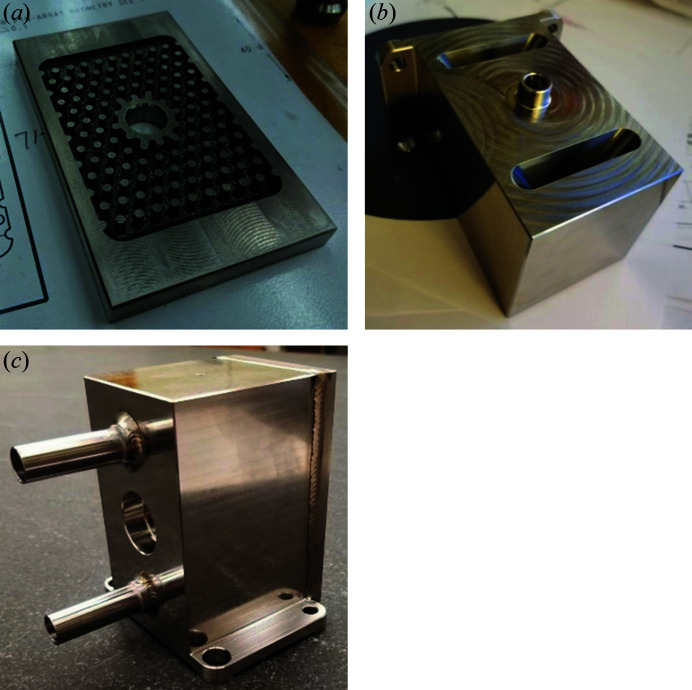
Coolant manifold machined from Invar 36 in separate pieces. The pin array (*a*) was made by electro-discharge machining, the block with the coolant passages was milled (*b*). These pieces were then welded together (*c*), heat-treated and the mirror–substrate interface surface was ground flat.

The coolant manifold was made from Invar 36 at the mechanical engineering shop at LBNL. Because the manifold contains many enclosed volumes – coolant passages, diffuser-shaped ducts and an array of pins – the manifold was made by welding separate pieces together (Fig. 2 ▸). One piece with the pin array was made by electro-discharge machining. A second piece containing the diffuser was made by milling. The coolant inlet and outlet were turned. After welding the manifold was heat-treated using the Lement process to achieve the required temporal and temperature stability (Lement, 1949 ▸) and then the mounting surface for the mirror substrate was ground to achieve the required flatness and roughness tolerances.

The coolant was pumped by an Axilon ChillAx cryocooler. Coolant lines between the inside of the vacuum chamber and the coolant manifold were fabricated at the LBNL mechanical engineering shop from 316 alloy stainless steel tubing. To facilitate assembly and to attenuate vibrations from the coolant lines and vacuum chamber to the manifold, the coolant lines included a section of flexible bellows manufactured by Osaka Rasenkan Kogyo Co. Ltd. This ‘clear flow flex’ tubing features an internal alumina braid which has the effect of reducing flow-induced vibrations, compared with a bare bellows (Yamazaki *et al.*, 2013 ▸).

**Figure 3:**
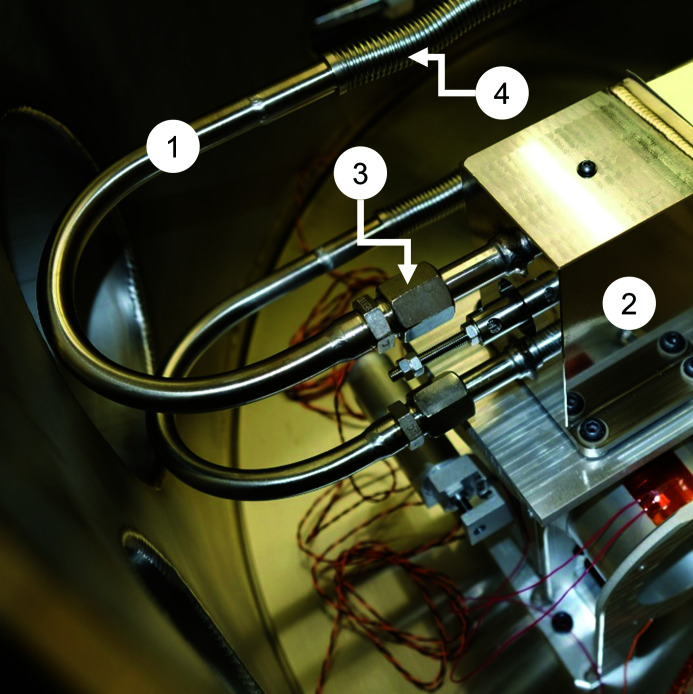
The liquid-nitrogen coolant lines (1) connect to the coolant manifold (2) with metal–face–seal fittings (3). The coolant lines include a section of flexible bellows with an internal braid (4).

The coolant lines were connected to the coolant manifold with metal–face–seal compression fittings (SERTO Straight Union Tube 12 mm SS316Ti 51021-12) (Fig. 3 ▸). These fittings were chosen for two reasons: (1) to explore alternatives to the Swagelok VCR fitting; but principally (2) to make a seal for liquid nitrogen between different materials: the 316 stainless steel coolant lines and the Invar coolant manifold.

Two alternative designs based on Swagelok VCR fittings included: (1) fabricating custom Swagelok VCR weld glands from Invar, which we did not pursue because the details of the Swagelok VCR gland are proprietary and it was not practical to measure and reverse-engineer them within our schedule constraints, and (2) welding standard off-the-shelf 316 stainless steel Swagelok VCR glands to the Invar coolant manifold. We did not pursue this option because of concerns that the mismatch in the thermal expansion of Invar and 316 stainless steel might cause cracks (and therefore leaks) to form during cooling from weld temperature to liquid-nitrogen temperature. However this issue can be mitigated by the design of the weld joint to minimize the welding heat needed as well as allowing sufficient flexibility in the joint once cooled to permit differential thermal expansion. In fact, this design strategy has been used successfully in liquid-nitrogen-cooled monochromators at Stanford Synchrotron Radiation Laboratory (Rowen *et al.*, 2001 ▸).

After assembly of the coolant lines to the coolant manifold, a helium leak check indicated a leak at the compression fittings between the manifold and coolant lines. The leak rate was approximately 4.5 × 10^−4^ Torr l^−1^ s^−1^. To determine the location and cause of the leak, we bench tested a series of fittings from the same production batch. These fittings showed an average leak tightness of 6 × 10^−10^ Torr l^−1^ s^−1^, which was more leak-tight than even the manufacturer specifications. The leak was fixed by injecting diluted vacuum epoxy into the fitting joint, and has subsequently been cycled between room and liquid-nitrogen temperature approximately 15 times without any sign of leaking. Therefore, we concluded that the leak was located not at the face seal, but between the internal diameter of the compression fitting and the outer diameter of the tubes welded to the coolant manifold. This leak may have been caused by distortion of the tube which could have occurred during welding or heat treatment. Because the parts were machined, welded and heat-treated within the specified tolerances, we would recommend that this problem needs to be addressed by a redesign of the welded joint.

To minimize handling of our prototype mirror substrate during initial assembly and fit checks, and therefore the risk of damage that would delay our schedule, we made a dimensional copy of the mirror in 6061 aluminium alloy. We used this aluminium ‘dummy’ mirror to check the basic fit of the various components in the vacuum chamber, and to rehearse the assembly of the mirror onto the coolant manifold. The aluminium dummy mirror also had tapped holes which facilitate the attachment of thermocouples. Separately, we tested the fit of the silicon mirror substrate to the Invar coolant manifold and the barrel nut clamping hardware. Testing indicated a need to rework both the coolant manifold and the aluminium dummy mirror. This rework consisted of removing a small (<5 g) amount of material from the manifold bore with a reamer, and increasing the diameter of the bored hole in the dummy mirror to fit the hollow dowel pin pressed into the manifold.

## 3. Fracture test

In the past, cryogenically cooled silicon monochromator crystals at the ALS have fractured during assembly. Therefore, we were motivated to test the fracture behavior of our prototype cryogenically cooled silicon mirror system. For a brittle material like silicon, the probability of fracture depends not only on the stress field but also on the dimensions and locations of surface defects such as cracks. Based on a fracture toughness value of 1 MPa m^1/2^ (Muhlstein *et al.*, 2001 ▸; St John, 1971 ▸; Ebrahimi & Kalwani, 1999 ▸; Chen & Leipold, 1980 ▸; Wong & Holbrook, 1987 ▸; Lawn *et al.*, 1981 ▸), we estimated that, in the most highly stressed region of the prototype mirror substrate, the maximum allowable crack length was 0.275 mm. This region is near the intersection of the holes for the barrel nut and tension rod, where the pressure applied to the substrate by the barrel nut through a layer of indium foil causes tensile contact stresses. The intersection of the holes is also an area where cracks can occur during fabrication, depending on the order in which the holes were drilled and the pressure on the drilling tool as it breaks through into the existing hole. Compared with the exterior surfaces of the mirror substrate, the insides of holes were difficult to inspect for defects. Stress in the substrate was – to a first order – proportional to the nominal clamping load. For example, the previously mentioned maximum allowable crack length of 0.275 mm corresponded to a clamping load of 750 N. It was anticipated that the clamping load would be a key parameter for the effective thermal conductivity and modal response of the system: increasing the clamping load would have the effect of increasing the effective thermal conductivity of the mirror substrate–cooling manifold interface, and increasing the first natural frequency of rigid body mode vibrations of the mirror substrate relative to the manifold.

**Figure 4:**
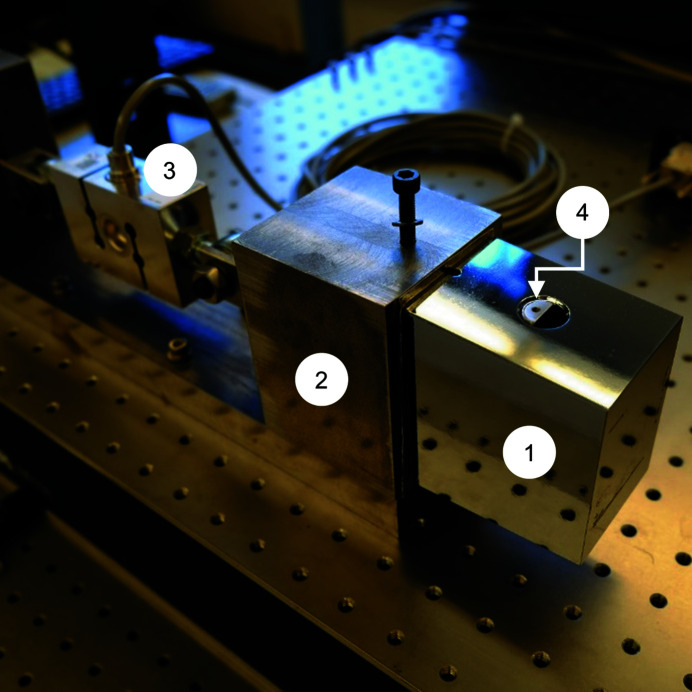
Fracture test apparatus consisting of the silicon test part (1), a steel block bolted to an optical table (2) and load cell (3), and the same barrel nut (4), indium foil and tension rod used in the prototype mirror system.

Given all this, we were motivated to test the robustness of our design. To permit the schedule advantages of testing in parallel, and to avoid breaking our prototype mirror, we fabricated a silicon test part for fracture testing. This silicon test was a truncated and unpolished version of the silicon mirror substrate, and was made by InSync, Inc. of Albuquerque, New Mexico, USA, using the same process as the prototype mirror substrate. Our test apparatus consisted of: the test part which we clamped to a steel block using the same barrel nut, a tension rod and indium foil hardware used with the prototype mirror substrate, and a calibrated load cell with an absolute accuracy of ±1 N to measure the clamp load (Fig. 4 ▸). To protect the personnel performing the test in the event of an energetic failure, we also built an impact-proof enclosure for the test part after testing the impact resistance of two samples of Lexan plastic sheet. After assembling the silicon sample onto the test apparatus, we increased the clamping load from a nominal 500 N at an average rate of 2 N s^−1^. We stopped the test when we observed yielding in the tension rod at a load of 2907 ± 1 N. At no point did we observe damage in the silicon test part. We conclude from this test that the silicon test part and therefore the mirror substrate can safely be loaded to at least 2907 ± 1 N without fracture, with the caveat that this conclusion is based on a single sample.

## 4. Heat-transfer tests

Two important assumptions in the design of this cryogenically cooled silicon mirror were the heat-transfer coefficients between the mirror substrate and the coolant manifold and between the coolant manifold and the liquid nitrogen (Cutler *et al.*, 2020 ▸). For design purposes, we assumed a value of 1500 W m^−2^ K^−1^ for the substrate–manifold interface based on published values for the thermal contact conductance (Asano *et al.*, 1993 ▸; Khounsary *et al.*, 1997 ▸; Marion *et al.*, 2004 ▸; Mochizuki *et al.*, 2007 ▸). For the manifold coolant–pin array interface we relied on the formula for forced convection developed by Žukauskas (1972 ▸). Although these studies provide useful information, practical questions about the heat-transfer coefficient during operation of the mirror system remained. Two such questions were: would the effective heat transfer coefficient vary with mirror temperature or with multiple thermal cycles between room and cryogenic temperature?

**Figure 5:**
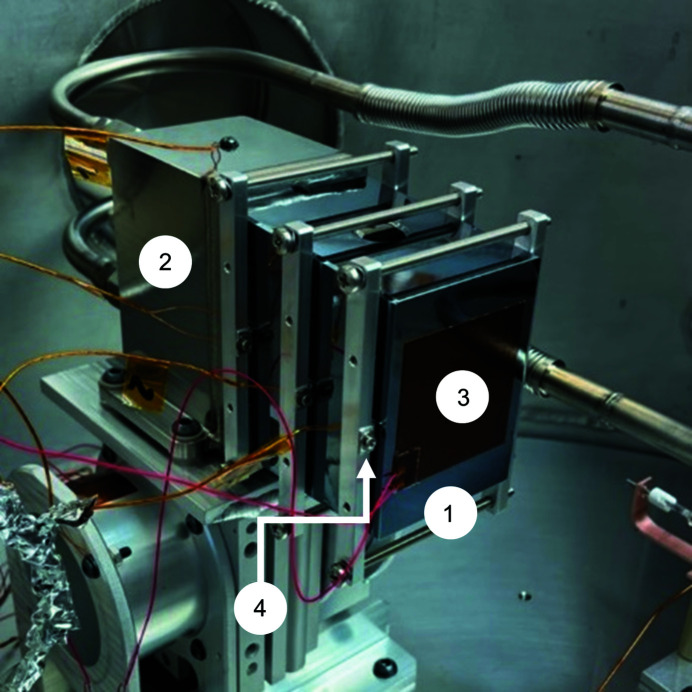
In the first heat-transfer test we clamped the silicon fracture test part or ‘short mirror’ (1) to the coolant manifold (2) and attached a film heater (3) and clamped thermocouples (4) to the mirror.

**Figure 6:**
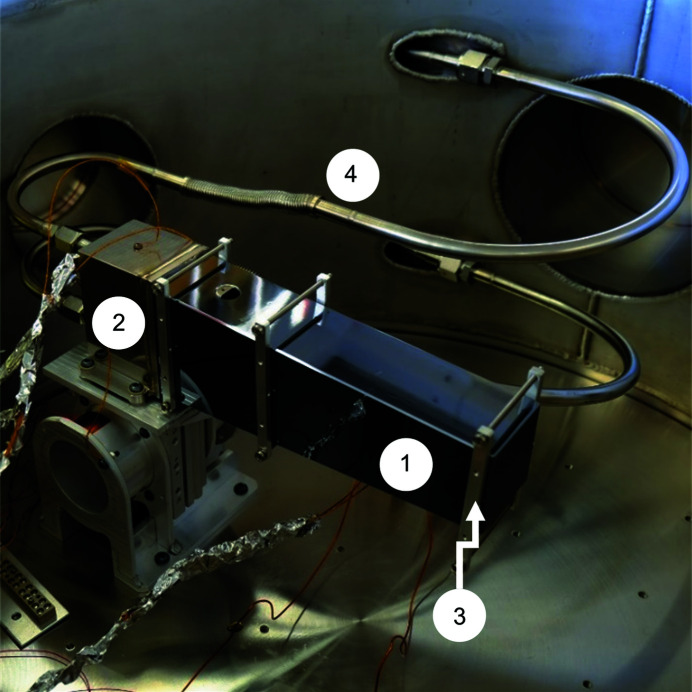
In the second heat-transfer test we clamped the silicon mirror substrate or ‘long mirror’ (1) to the coolant manifold (2) and attached thermocouples (3). The coolant lines (4) and the flexible bellows are also visible.

To check the validity of our assumptions and evaluate the cyclic thermal behavior of the system we created two heat-transfer tests. In the first test, we assembled the fracture test part onto the coolant manifold (Fig. 5 ▸). For ease of reading in this section we will refer to the fracture test part as the ‘short mirror’. We placed a 0.5 mm-thick layer of indium foil between the short mirror and the manifold. In the second test, we replaced the short mirror with the prototype mirror (Fig. 6 ▸), which we will refer to in this section as the ‘long mirrr’. The same piece of indium foil was reused. The clamp torques were 2 N m for the short mirror and 2.5 N m for the long mirror. During both tests we recorded the temperature of the mirror, the manifold and the inside of the vacuum chamber while cycling the manifold temperature between 285 ± 10 K and 87 ± 1 K. We cooled the system with liquid nitrogen and warmed the system with gaseous nitrogen.

We then calculated the effective heat-transfer coefficient from the experimental data using a lumped mass model for the energy balance of the mirror,

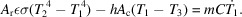

There are two modes of heat transfer in this model: (1) radiation from the vacuum chamber to the mirror and (2) conduction from the mirror substrate to the manifold combined with convection from the manifold to the liquid nitrogen. The radiation term is composed of the area of the mirror that absorbs radiation *A*_r_, the emissivity ε, the Stefan–Boltzmann constant σ, the temperature of the vacuum chamber *T*_2_ and the temperature of the mirror *T*_1_. The conduction and convection term is the product of the effective heat-transfer coefficient *h*, the effective interface area *A*_c_, and the difference between the mirror temperature *T*_1_ and nitrogen temperature *T*_3_. In other words, with these modeling assumptions, the effective heat-transfer coefficient combines three steps in the heat-transfer path: the thermal contact conductance of the silicon–indium–Invar interface, conduction in the Invar heat exchanger and convection from the pin array in the heat exchanger to the nitrogen. The measured effective heat-transfer coefficient is therefore different to the coefficients cited above and used during the design phase, which are just for the thermal contact conductance (Asano *et al.*, 1993 ▸; Khounsary *et al.*, 1997 ▸; Marion *et al.*, 2004 ▸; Mochizuki *et al.*, 2007 ▸) or forced convection (Žukauskas, 1972 ▸). The net power transferred to the mirror substrate is the product of the mirror mass *m*, its specific heat *C* and the time rate of change of the its temperature 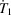 . Note that the model assumes radiative heat transfer between two bodies: the mirror and the vacuum chamber. In reality the radiative environment has additional parts, for example coolant lines and chamber windows. The temperatures, view factors, areas and emissivities of these additional parts were not included in the lumped mass model because the overall radiative exchange to the mirror is dominated by the relatively large area, emissivity and view factor of the vacuum chamber.

We calculated the areas *A*_r_ and *A*_c_ and volume from the mirror CAD-models; uncertainty in these values is related to the manufacturing tolerances of the actual parts. For the material properties of silicon, we assumed a density of 2330 kg m^−3^ (Peuto *et al.*, 1993 ▸; Mizushima *et al.*, 2004 ▸), a specific heat capacity of 707 J kg^−1^ K^−1^ (Touloukian & Buyco, 1971 ▸) and a temperature-dependent emissivity (Constancio Jr *et al.*, 2020 ▸).

**Figure 7:**
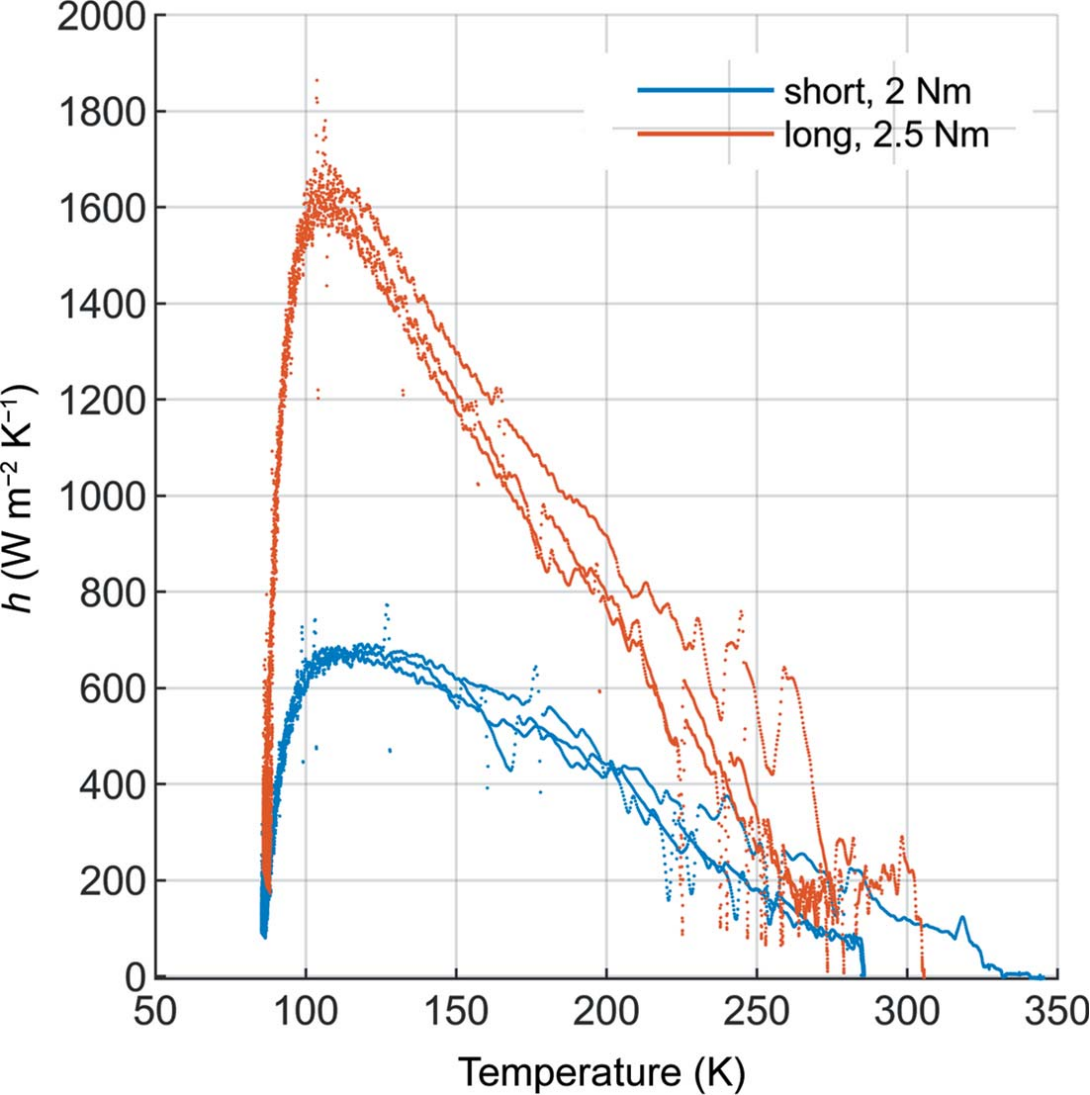
We observed a temperature dependence of the effective heat-transfer coefficient *h* for both the short and the long mirrors. In both cases the temperature of the peak *h* was approximately 106 K. The clamping torque was 2.5 N m for the long mirror and 2 N m for the short mirror. Data from multiple thermal cycles are plotted, showing the relatively large cycle-to-cycle variation in *h* above 210 K.

**Figure 8:**
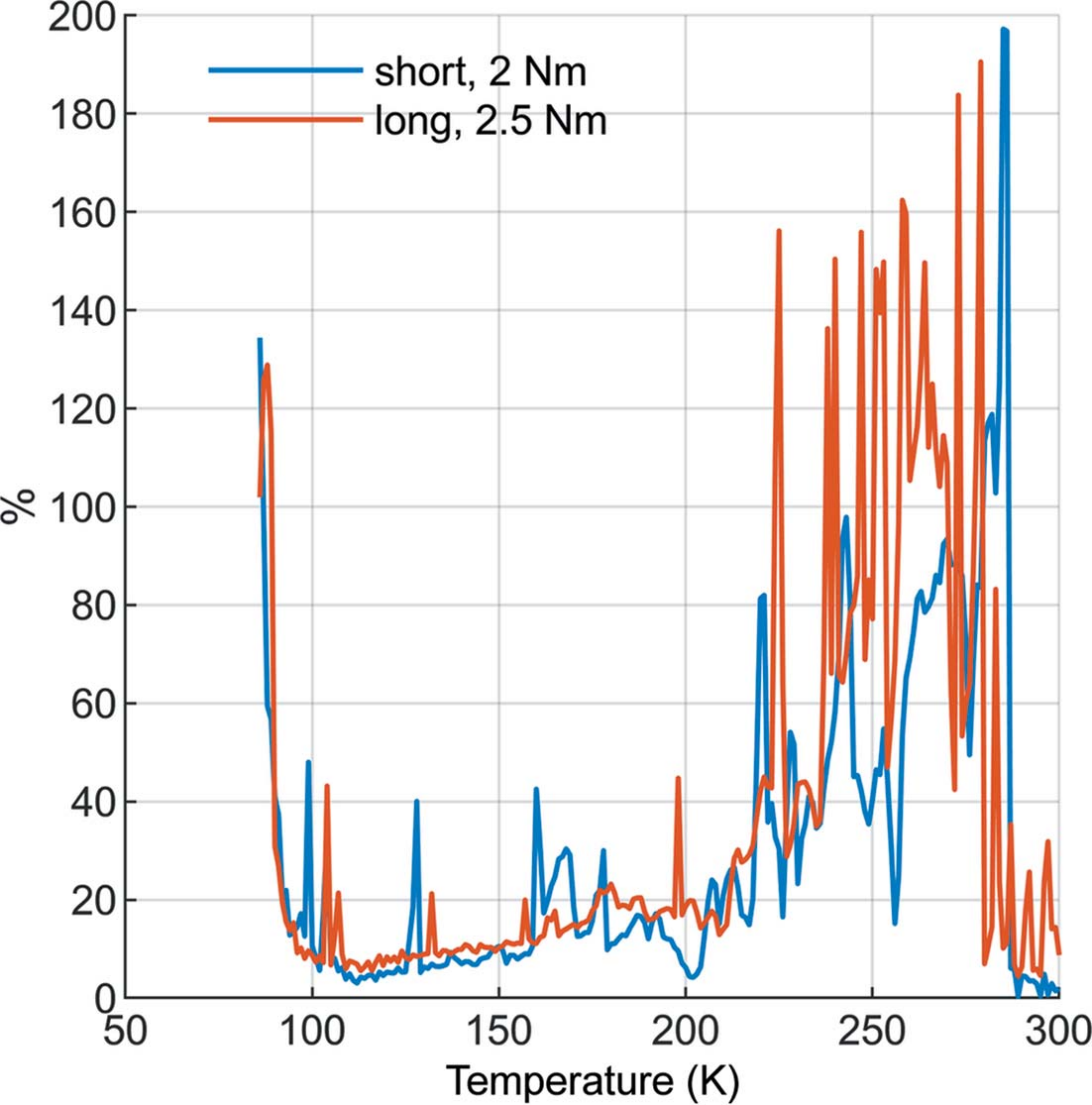
Cycle-to-cycle variability of the effective heat-transfer coefficient, which was less than 20% between 100 K and 210 K, with significantly greater variation outside this range.

With these assumptions and the experimental temperature data we calculated the effective heat-transfer coefficient *h*. We observed that *h* depended on the initial clamping torque and mirror temperature (Fig. 7 ▸). The peak value of *h* is approximately 1600 W m^−2^ K^−1^ at 106 K for the long mirror and also 700 W m^−2^ K^−1^ at 106 K for the short mirror. Cycle-to-cycle variation in *h* is less than 20% between 100 K and 225 K, and peaks at 200% and 290 ± 5 K (Fig. 8 ▸).

The observed temperature dependence of the effective heat-transfer coefficient *h* is the expected result of two factors: the temperature dependencies of (1) the substrate–manifold interface contact pressure and (2) thermal conductivity. The substrate–manifold interface contact pressure varies as the components in the ‘clamping chain’ expand or contract with temperature at different rates. These components are the silicon mirror substrate, Invar manifold, 316 stainless steel tension rod and spring washers, and indium foil between both the substrate and manifold and between the barrel nut and substrate. Because the calculated effective heat-transfer coefficient *h* combines both convection and conduction it is a function of the temperature-dependent thermal conductivities of the ‘heat-transfer chain’ made up of the substrate, indium foil and manifold.

## 5. Modal response

**Figure 9:**
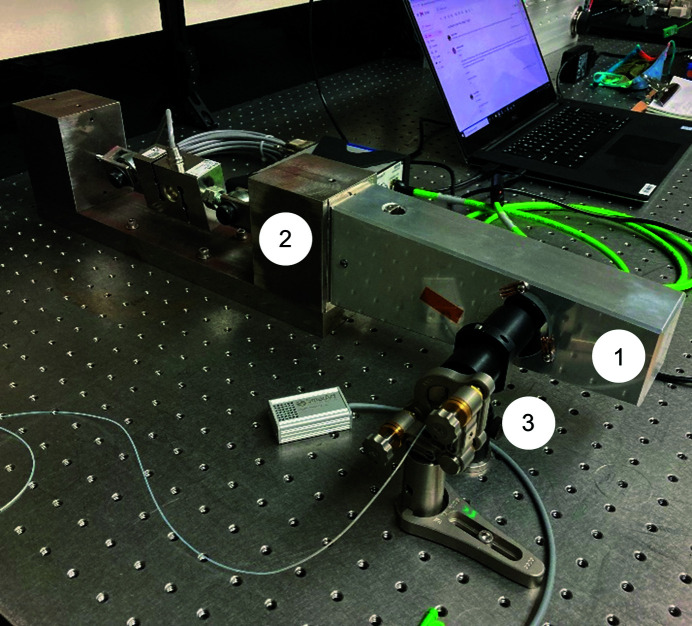
We measured the modal response of the aluminium dummy mirror (1) clamped to the fracture rig (2) using an interferometric distance sensor (3).

Mirror vibration is a problem because it can mis-steer the beam and cause downstream intensity variations. The performance of the beamline is particularly sensitive to movement of the mirror in the pitch direction, or rotation about the vertical axis coincident with the center of the reflecting surface. Given the ground motion of the current ALS and the beamline performance targets of the ALS-U, a modal response requirement has been derived for ALS-U M1s. Specifically, the natural frequencies of modes that have a pitch component are required to be above 200 Hz. The vibration of the cantilevered mirror substrate was modeled early in the design phase, and we estimated a first natural frequency of approximately 410 Hz. This estimate was based on assumptions about the indium layer, the tension rod, the barrel nut, and the indium foil between the barrel nut and the substrate. To evaluate the validity of these assumptions we experimentally measured the modal response of the aluminium dummy mirror clamped to the fracture test rig using an interferometer-based distance sensor (Fig. 9 ▸). We used an interferometer instead of an accelerometer for two reasons: to simplify identifying motion in the pitch direction and to measure the relative movement between the mirror and its mounting system. Noise in the interferometer system generally increases with the nominal measurement distance. At the approximately 100 mm distance used for these measurements, the RMS noise is 0.11 nm in the frequency decade 1–10 kHz (SmarAct, 2022 ▸); displacement measurements were on the order of 1 µm.

We used the aluminium dummy mirror instead of the silicon prototype so that we could carry out modal testing and cryogenic-deformation measurements in parallel. Because the density of 6061 aluminium is 16% higher than that of silicon, the measured natural frequencies of the aluminium dummy mirror are conservatively lower than that of the prototype silicon substrate. We also assumed that the modal response of the cantilevered mirror was dominated by the mirror–coolant manifold interface. Since we assumed that the mirror vibrated as a rigid body, we could neglect the difference in the elastic moduli of aluminium and silicon. Because the specific stiffness (elastic modulus/density) of silicon is approximately 2.2× higher than that of 6061 aluminium, the actual silicon mirror system will have higher natural frequencies than the aluminium dummy mirror for modes that contain elastic displacement of the mirror.

**Figure 10:**
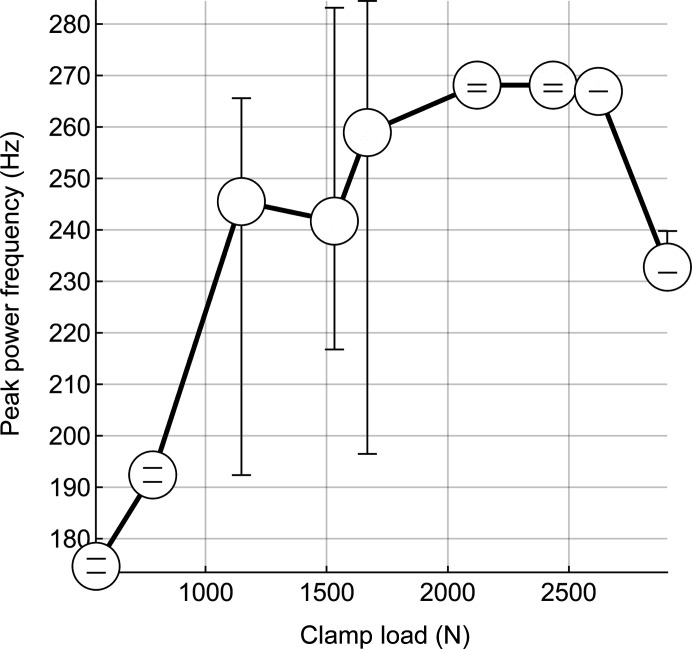
We observed that the peak power frequency increased with clamp load between 500 N and 2000 N. Above 2000 N, the modal response of the mirror system appeared to be dominated by a motion that is not a function of clamp load.

To excite vibrations in the mirror we applied an impulse in the pitch direction and recorded the displacement of the mirror. This process was repeated for a range of clamping loads between 500 N and 3000 N. After averaging the power spectral densities for each clamp load, we plotted the frequency at the peak power density versus clamp load (Fig. 10 ▸). We conclude that the modal response requirement can be met for clamping loads above 900 N. We observed that the peak power frequency of pitch modes increased with clamp load between 500 N and 2000 N, with significant measurement-to-measurement variation at intermediate clamping loads between 1200 N and 1700 N. Between 2000 N and 2600 N, the modal response of the mirror system appeared to be dominated by a pitch motion which did not depend on clamping load. Further increasing the clamping load to 3000 N caused the peak power frequency to decrease again, possibly because of yielding and related section reduction in the tension rod or the inelastic properties of the indium layer.

## 6. Mirror distortion

In our cryocantilever design, the mirror substrate is cantilevered to minimize distortion of the mirror. To evaluate the performance of this cantilevered design concept we measured the distortion of the mirror at various key points in its fabrication, assembly and operation. In this section we describe our distortion measurements and the development of our measurement system. In particular, we detail our efforts to distinguish between mirror distortion and the effects of the metrology window.

**Figure 11:**
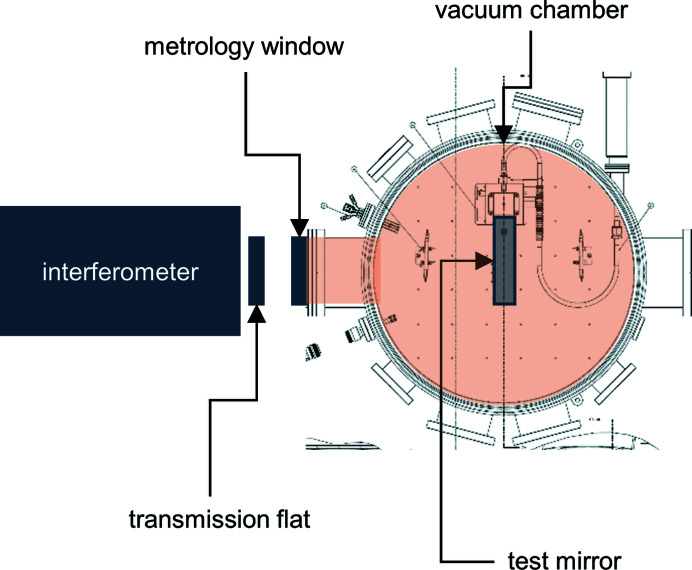
We measured the distortion of the mirror at both room and cryogenic temperatures using a Fizeau interferometer. The mirror was inside a vacuum chamber and the interferometer was in air. Measurements were therefore made through a metrology window.

Our mirror distortion measurement system was based on a Fizeau interferometer. The interferometry beam passed through a transmission flat and was reflected by the test mirror. The test mirror was inside a vacuum chamber, and was aligned to the interferometer using an in-vacuum optical tip-tilt stage mounted between the coolant manifold and the vacuum chamber base. Both the vacuum chamber and the interferometer were supported on a vibration-isolating optical table. As we will describe, we typically used a metrology window on the vacuum chamber located between the transmission flat and the mirror (Fig. 11 ▸).

The first step in the mirror measurements took place at InSync Inc. where the mirror was fabricated. After polishing, InSync measured an RMS height error of 8 nm (J. Metz, personal communication). This measurement was made with the mirror simply supported on its side, not cantilevered. This RMS height error value is for the entire mirror area except for a 5 mm-wide margin around the edges.

**Figure 12:**
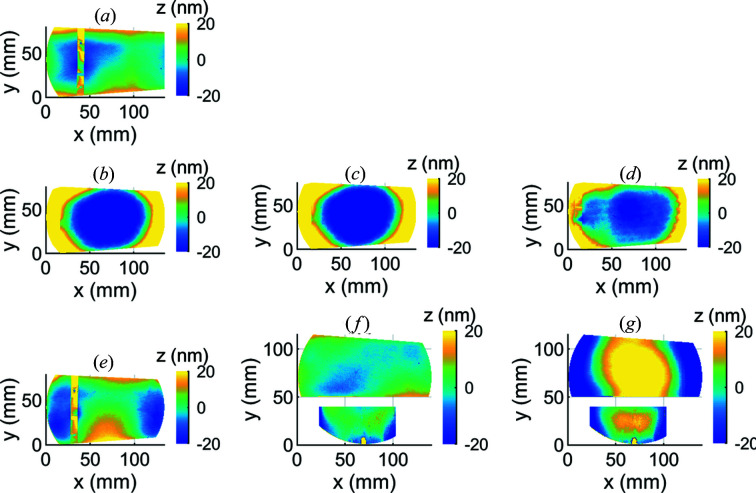
We measured the height error of the mirror after (*a*) assembly with the manifold and without a metrology window, (*b*) installation of a 9.4 mm-thick λ/4 window, (*c*) pumping down the vacuum chamber to 10^−8^ Torr, (*d*) cooling the mirror to 87 K, (*e*) switching the metrology window to a 25.4 mm-thick λ/20 window, (*f*) pumping down the vacuum chamber to 10^−8^ Torr, (*g*) cooling the mirror to 98.75 K. Note that the measurements shown in (*a*) and (*e*) were made with a thermocouple clamped to the mirror substrate; the clamp is visible as a vertical band. Note also that the measurements shown in (*f*) and (*g*) include a reference mirror to separate window effects from mirror height error.

The second measurement took place at LBNL, where we assembled the test mirror to the coolant manifold and consequently measured an average RMS height error of 7 ± 1 nm [Fig. 12 ▸(*a*)]. This measurement was made with a clamping torque of (2 ± 0.06) N m and without a metrology window. Compared with the measurement made at InSync before assembly, this measurement was made on a smaller portion of the mirror: the portion visible through the nominal 150 mm-diameter vacuum chamber port. With this caveat, we did not observe any distortion attributed to assembly.

Next, we installed a metrology window on our vacuum chamber. This window was made from fused silica, had a view diameter of 136 mm, a thickness of 9.4 mm and was polished flat to a peak-to-valley height error tolerance of λ/4 (158 nm). The effect of this window on the apparent distortion of the mirror was a concave-spherical shape with an RMS height error of 32 ± 1 nm [Fig. 12 ▸(*b*)]. This shape remained after we pumped the vacuum chamber down to a nominal 10^−8^ Torr [Fig. 12 ▸(*c*)] and also after we cooled the mirror to 87 ± 0.34 K [Fig. 12 ▸(*d*)]. Therefore, we concluded that the window affected the accuracy of our measurement, and that this effect was not strongly related to the pressure difference between the inside and outside of the window.

To better understand the effect of the window on the measurement, we replaced the 9.4 mm-thick λ/4 window with a dual-surface λ/20 optical flat, made from fused silica with a nominal diameter of 152.4 mm and thickness of 25.4 mm. Care was taken in the design and assembly of this window so that the glass was in contact only with rubber o-rings and that reaction forces of the o-rings were aligned to minimize bending moments in the glass. We repeated the measurement with the chamber at atmospheric pressure [Fig. 12 ▸(*e*)] and pumped down to 10^−8^ Torr [Fig. 12 ▸(*f*)]. The corresponding RMS height errors were 9 ± 1 nm at atmospheric pressure and 6 ± 1 nm with the chamber pumped down. Compared with the first window, the measurements made through the second window more closely match the measurement made without any window.

To help distinguish window effects from mirror distortion, we installed a second reference mirror inside the vacuum chamber. This reference mirror was made from low-thermal-expansion glass and was placed unconstrained on the vacuum chamber floor to minimize strain while also providing sufficient thermal contact to maintain the reference mirror at approximately the same temperature as the vacuum chamber and ambient air temperature. Using the λ/20 window, and with the vacuum chamber pumped down to 10^−8^ Torr, we simultaneously measured the distortion of both the test and the reference mirrors at a temperature of 293 ± 0.44 K [Fig. 12 ▸(*f*)]. The RMS height errors of the test and reference mirrors were 7 ± 1 nm and 16 ± 1 nm, respectively.

We then cooled the test mirror and repeated the measurements. Average mirror temperatures during the measurement period were 98.75 ± 0.35 K for the test mirror and 293 ± 0.44 K for the reference mirror. Both the test and the reference mirrors appeared to become convex and spherical [Fig. 12 ▸(*g*)]. The RMS height errors of the test and reference mirrors were 39 ± 1 nm and 28 ± 1 nm, respectively. Because we removed separate best-fit planes from the raw measurement data to correct for the imperfect alignment of the mirrors to the interferometer, when plotted the center of the spherical error appears to shift from the center of the window towards the centers of the individual mirrors.

Because both the test and the reference mirrors appeared to change shape as the test mirror was cooled, we suspect that the temperature-dependent properties of the metrology window were affecting the measurement. For fused silica at room temperature, the temperature coefficient of the refractive index is 8.65 ± 0.18 p.p.m. K^−1^ (Dupouy *et al.*, 2010 ▸; Malitson, 1965 ▸; Matsuoka *et al.*, 1991 ▸). We estimated that when the air side of the metrology window was 295 K and the test mirror was 87 K, the vacuum side of the metrology window was 290 K due to radiative heat transfer from the metrology window to the test mirror.

## 7. Conclusions

Through the testing described in this report we concluded that the cryocantilever prototype functions as intended. At the time of writing, the prototype has been thermally cycled between room and cryogenic temperature approximately 15 times without any observed change in behavior. Suggestions for future work would include measuring the distortion of the mirror with a heat load. Because the accelerator and beamline upgrades of the ALS-U have not been built yet, an alternative source for this heat load could be an industrial cutting laser. This high-power laser would also be useful for improving the accuracy of heat-transfer measurements by increasing the scale of temperature differences throughout the system. A related topic that merits further research is metrology of cryogenic optics. As discussed, the interferometric measurement of nanometre-scale height errors through a vacuum chamber window is complicated by radiative heat transfer between the window and the test mirror. Developments in this area are potentially interesting not only to the synchrotron radiation light source community but also to people working on space-based telescopes (Kegley *et al.*, 2006 ▸).
